# LncRNA and mRNA expression characteristic and bioinformatic analysis in myocardium of diabetic cardiomyopathy mice

**DOI:** 10.1186/s12864-024-10235-z

**Published:** 2024-03-26

**Authors:** Mengnan Zhao, Ting Wang, Xiaoning Cai, Guizhi Li, Na Li, Hong Zhou

**Affiliations:** 1 Department of Endocrinology, The Second Hospital of Hebei Medical University, No. 215, Hepingxi Road, Xinhua District, 050000 Shijiazhuang, Hebei China; 2 Department of Endocrinology, Liaocheng Traditional Chinese Medicine Hospital, No. 1, Wenhua Road, Dongchangfu District, 252000 Liaocheng, Shandong China

**Keywords:** Long non-coding RNA, Expression profile, Diabetic cardiomyopathy, lncRNA-mRNA co-expression, Bioinformatic analysis, Transcriptome sequencing

## Abstract

**Background:**

Diabetic cardiomyopathy (DCM) is becoming a very well-known clinical entity and leads to increased heart failure in diabetic patients. Long non-coding RNAs (LncRNAs) play an important role in the pathogenesis of DCM. In the present study, the expression profiles of lncRNAs and mRNAs were illuminated in myocardium from DCM mice, with purpose of exploring probable pathological processes of DCM involved by differentially expressed genes in order to provide a new direction for the future researches of DCM.

**Results:**

The results showed that a total of 93 differentially expressed lncRNA transcripts and 881 mRNA transcripts were aberrantly expressed in db/db mice compared with the controls. The top 6 differentially expressed lncRNAs like up-regulated Hmga1b, Gm8909, Gm50252 and down-regulated Msantd4, 4933413J09Rik, Gm41414 have not yet been reported in DCM. The lncRNAs-mRNAs co-expression network analysis showed that LncRNA 2610507I01Rik, 2310015A16Rik, Gm10503, A930015D03Rik and Gm48483 were the most relevant to differentially expressed mRNAs.

**Conclusion:**

Our results showed that db/db DCM mice exist differentially expressed lncRNAs and mRNAs in hearts. These differentially expressed lncRNAs may be involved in the pathological process of cardiomyocyte apoptosis and fibrosis in DCM.

## Background

Diabetes mellitus (DM) has become a global public health problem. The people with diabetes have a higher chance of developing heart failure (HF) than those without diabetes [1]. Diabetic cardiomyopathy (DCM) is becoming a very well-known clinical entity, which increase the risk of hospitalization for HF and the mortality of patients with type 2 diabetes mellitus (T2DM) [2]. DCM is characterized by cardiomyocyte apoptosis, hypertrophy and myocardial fibrosis, leading to firstly diastolic dysfunction, later systolic dysfunction and eventually clinical HF, independent of coronary artery disease, hypertension or valvular heart diseases [3, 4]. In recent years, increasing evidence demonstrate that insulin resistance, oxidative stress, mitochondrial dysfunction, maladaptive immune responses and impaired calcium homeostasis are related to the development of DCM [5, 6]. Changes in gene regulation such as activation of transcription factors, microRNAs (miRNAs) and epigenetic mechanisms have also been implicated in the progression of DCM [7, 8]. Although a variety of underlying pathogenesis have been confirmed to be associated with DCM, no specific therapeutic method has yet been established to prevent the progression of DCM. Thus, there is an urgent need to further explore the biological markers and clear therapeutic targets at early stage of DCM.

Long non-coding RNA (lncRNA), a type of ‘regulatory’ RNAs of non-coding RNAs, which is mainly transcribed by RNA polymerases II in mammal animals and is greater than 200 nucleotides in length but lack of protein-coding capacity [9, 10]. With the development of high throughput RNA-sequencing (RNA-seq), thousands of lncRNAs have been identified and many potential functions of lncRNAs have been discovered. Based on their genomic proximity to protein coding genes, lncRNAs can be categorized as sense, antisense, intronic, intergenic and bidirectional lncRNAs [11]. Although the functions of the majority of lncRNAs remains to be fully elucidated, the established functions include functioning as scaffolds, modulating protein activity and regulating gene expression containing chromatin modification, regulation of transcription initiation and co- and post-transcriptional regulation [10, 12]. Recently, studies show that abnormal lncRNA expression may contribute to the development and progression of DCM [13–16]. Moreover, lncRNAs can also act as competitive endogenous RNAs (ceRNAs) to compete with target genes for miRNAs to influence the course of DCM [17, 18], including NLRP3 inflammasome activation-mediated pyroptosis [19], cardiac fibrosis and hypertrophy [20, 21], as well as cardiomyocyte apoptosis by epigenetic regulation of target genes [22]. However, few studies have comprehensively drawn the expression characteristics of lncRNAs in DCM, and the lncRNA-related regulation mechanism in DCM is yet to be clarified.

In the present study, we established DCM mouse models and obtained differentially expressed lncRNAs and mRNAs in heart tissues, and analyzed some possible pathological processes of DCM involved by differentially expressed genes in order to provide a new direction for the future researches of DCM.

## Methods

### Animal model

The animal study was approved by the Medical Ethics Committee of the Second Hospital of Hebei Medical University and comply with the ARRIVE guidelines. 8-week-old obese male BKS.Cg-Dock7^m^+/+Lepr^db^ (db/db) mice weighing 40–42 g were purchased from Nanjing Junke Bioengineering Co., Ltd. (Nanjing, China) used as experimental group and age matched C57BL/6J male mice weighing 19–21 g from the same company were used as control group. All db/db mice random blood glucose ≥ 16.7 mmol/L, which were considered as animal models of T2DM. Mice were housed under controlled conditions at 22–26℃ with 12-hour light/dark cycles and 40–70% humidity at the standard mice cages in the laboratory animal center of the Second Hospital of Hebei Medical University. Ensure that the laboratory is completely clean and that the mice have free access to food and water during the experiment. Body weights (BW) were measured weekly and the blood glucose was measured by a blood glucose meter (Roche, Germany) from the tail vein. Systolic arterial blood pressure (SABP) was measured monthly by tail-cuff micro-photoelectric plethysmography. At 20 weeks of age, all biochemical index were measured using the automatic biochemical instrument of the Second Hospital of Hebei Medical University, and serum insulin levels was performed using an ELISA kit (Senberga, Nanjing, China) according to the manufacturer’s instructions. Insulin resistance index (HOMA-IR) was calculated as FBG×FINS/22.5 and was used to assess insulin resistance.

### Echocardiographic examination

At 20 weeks of age, echocardiography was performed using a 11-MHz linear transducer coupled to a high-resolution Ultrasound System (vivid E95, GE Healthcare, USA,) to evaluate cardiac structure and function in mice. Compared to non-volatile anesthetics, the anesthetic isoflurane has less effect on systemic hemodynamics and better preserves cardiac function in mice [23]. Therefore, isoflurane was chosen as the anesthetic agent with an induction concentration adjusted to 2-2.5% and a maintenance concentration of 1-1.5%. Mice were anesthetized using a RWD small animal anesthesia machine (Shenzhen, China, model R500). Serial M-mode echocardiographic images were taken in the short axis view at the level of the papillary muscles. Indicators representing left ventricular systolic function were obtained: Left ventricular end-diastolic diameter (LVEDD), left ventricular end-systolic diameter (LVESD), left ventricular end diastolic volume (EDV) and end systolic volume (ESV), Left ventricular fractional shortening (LVFS) and left ventricular ejection fraction (LVEF). Subsequently, the mitral peak flow velocities at early diastole (E) and atrial contraction (A) were detected by pulsed Doppler technique. Tissue Doppler imaging was obtained from the lateral mitral valve annulus, and mitral annulus early diastolic velocity(E′)were measured. E/A and E/E′ ratios were calculated to reflect left ventricular diastolic function. All measurements were averaged for three consecutive cardiac cycles by an experienced technician.

### Histological examination and TUNEL assay

At 20 weeks of age, the mice were executed and heart tissues located in the left ventricle were removed and fixed in 4% paraformaldehyde for 24 h at room temperature and then embedded in paraffin. Specimen sections that were cut into 5 μm thicknesses were stained with Masson’s trichrome and hematoxylin and eosin (H&E), and then observed the morphology of the cardiomyocytes and the deposition of collagen under an optical microscope. Apoptosis of cardiac myocytes was determined using the terminal deoxynucleotidyl transferase-mediated dUTP nick-end labeling (TUNEL) assay.

### RNA-Sequencing and bioinformatic analysis

Heart tissues were collected quickly into enzyme-free cryogenic vials after aspirating the surface liquid. They were snap frozen in liquid nitrogen and then transferred to -80 °C for storage. Subsequently, total RNA was extracted from heart tissues. The concentration and purity of RNA were examined using Nanodrop2000, RNA integrity was measured by agarose gel electrophoresis, and RNA integrity number (RIN) values were determined by Agilent 2100. The samples with RIN ≥ 8.0, OD260/280 ≥ 1.8, OD260/230 ≥ 1.0 were subjected to subsequent analysis. Sequencing experiments were performed with the help of Shanghai Majorbio Bio-pharm Technology Co.,Ltd. Expression levels of transcripts were quantified using RSEM (Version 1.2.31) [24]. Transcripts Per Million reads (TPM) was used as a quantitative metric, and transcript length and sequencing depth were homogenised, thus obtaining normalised expression levels of lncRNAs and mRNAs. Differential expression analysis were performed using DESeq2 (Version 1.10.1), and P-adjust < 0.05 &|log2FC|>=1 were used as screening criteria to obtain differentially expressed transcripts [25].

### Analysis of the lncRNA-mRNA co-expression network

Differentially expressed lncRNAs and mRNAs obtained by sequencing were used to construct a lncRNAs-mRNAs co-expression network with the aim of screening for potential targets of lncRNAs. The inclusion conditions for lncRNA-mRNA pairs are Pearson correlation coefficient (PCC) value > 0.99, PCC<-0.99 and *P* < 0.05. Cytoscape v3.9.1 was used to graphically present the network where each transcript corresponds to a node and the lines between the nodes indicate that they have strong correlation.

### GO and KEGG enrichment analysis

Gene Ontology (GO) and Kyoto Encyclopedia of Genes and Genomes (KEGG) pathway enrichment analyses were used to assess the potential biological function of the genes. GO database can describe the function of genes and proteins. Therefore, The function of differentially expressed genes were analyzed in terms of biological process (BP), cellular component (CC) and molecular function (MF). KEGG database were used to classify the differentially expressed genes by the pathway and function they were involved.

### Real-time quantitative PCR

To validate the expression profile data obtained from RNA-seq, we selected six lncRNAs which with significant changes for validation using real-time quantitative PCR. Total RNA from the heart of mice (*n* = 3) was isolated as the manufacturer’s guidelines using RNA-easy™ Isolation Reagent (Vazyme, Nanjing, China). The concentration and purity of RNA were examined using Nanodrop2000 (Thermo Scientific, Waltham, MA, USA). And then, total RNA was processed by reverse transcription reactions with a Thermo Scientific™ RevertAid™ First Strand cDNA Synthesis Kit (Thermo Scientific, Waltham, MA, USA). GAPDH was used as the internal reference. The expression levels of lncRNAs were determined by CFX96^TM^Real-Time System (Bio-Rad, California, USA) using GOTaq® qPCR Master Mix (Promega, Beijing, China). The reaction conditions were as follows: pre-denaturation at 95 °C for 10 min, 45 cycles of denaturation at 95 °C for 15 s, and annealing at 60 °C for 1 min. The primers are listed in Table 1. The relative expression of the target gene was calculated using the 2^−ΔΔCt^ method. All experiments were repeated three times.

**Table 1:** The primer sequences designed for PCR

Type	Transcript id	Primer
lncRNA	ENSMUST00000214387	Forward: CGGGACCAGGACTGAGGACTTAG Reverse: TCGCATTGTCAGAGAACTGGGAAAG
lncRNA	ENSMUST00000219113	Forward: GCCGCCACTAGATGGTGCTAAAC Reverse: CCTCGGAGACGGACTGGTGAC
lncRNA	ENSMUST00000060680	Forward: AATTTGTCCCGTCTTGTGCAGAGG Reverse: CGCCATCAACCCCAGATTCGTG
lncRNA	ENSMUST00000136201	Forward: TGGCTAACGAGTTGTGTTCCTCTTC Reverse: AGTTGGTGGCTTGGCTGCTTG
lncRNA	NR_037964.1	Forward: TCACTATGGAGGGCAGGGAAGC Reverse: TGAAGAGGCTAAGGCAGGAGGATC
lncRNA	chr15:78850587–78,850,791	Forward: CAGATCACCGGGAGGGAGACAG Reverse: AGGAAACTGAGGCACGGGGTATAG

### Statistical analysis

All data were expressed as mean ± SD, and the statistical analysis of different groups was performed using Student’s t test in SPSS (Version 21.0). graphs were drawn using GraphPad Prism (Version 8.0.1) and R software (Version 4.1.3). The value of *P*<0.05 (two tailed) was considered statistically significant.

## Results

### General characteristics of animals

At the age of 20 weeks, The ratio of heart weight to body weight (HW/BW), fasting blood glucose (FBG), serum insulin (INS), Hemoglobin A1c (HbA1c), serum total cholesterol (TC) and alanine aminotransferase (ALT) levels as well as HOMA-IR were all remarkably higher in db/db than those in control mice (*p*<0.01). Thus, db/db mice exist hyperglycemia, high fat and insulin resistance. No significant differences were detected in SABP, triglyceride (TG), low-density lipoprotein cholesterol (LDL-C) and creatinine (CRE) levels between the groups (*p*>0.05). The plasma parameters were shown in Table 2.

**Table 2:** Plasma biochemical parameters, HW/BW and SABP (*n* = 6)

	Control group	db/db group
FBG, mmol/L	5.30 ± 0.79	31.22 ± 6.25^**^
HbA1c, %	4.58 ± 0.37	9.40 ± 1.04^**^
INS, mU/L	73.98 ± 10.15	314.30 ± 46.53^**^
HOME-IR	17.37 ± 3.26	438.69 ± 118.66^**^
TC, mmol/L	2.53 ± 0.27	4.17 ± 0.76^**^
TG, mmol/L	1.09 ± 0.09	1.45 ± 0.83
LDL-C, mmol/L	0.22 ± 0.07	0.36 ± 0.15
ALT, U/L	35.21 ± 3.22	126.68 ± 62.42^**^
CRE, µmol/L	11.63 ± 1.51	9.54 ± 1.77
HW/BW (×10^− 3^)	5.12 ± 0.14	5.79 ± 0.21^**^
SABP, mmHg	121 ± 5	123 ± 7

^**^P<0.01 compared with control group.

Notes: FBG: fasting blood glucose; HbA1c: Hemoglobin A1c; INS: serum insulin; HOMA-IR: Insulin resistance index; TC: serum total cholesterol; TG: triglyceride; LDL-C: low-density lipoprotein cholesterol; ALT: alanine aminotransferase; CRE: creatinine; HW/BW: the ratio of heart weight to body weight; SABP: systolic arterial blood pressure.

### Establishment of DCM models

**Fig. 1:**
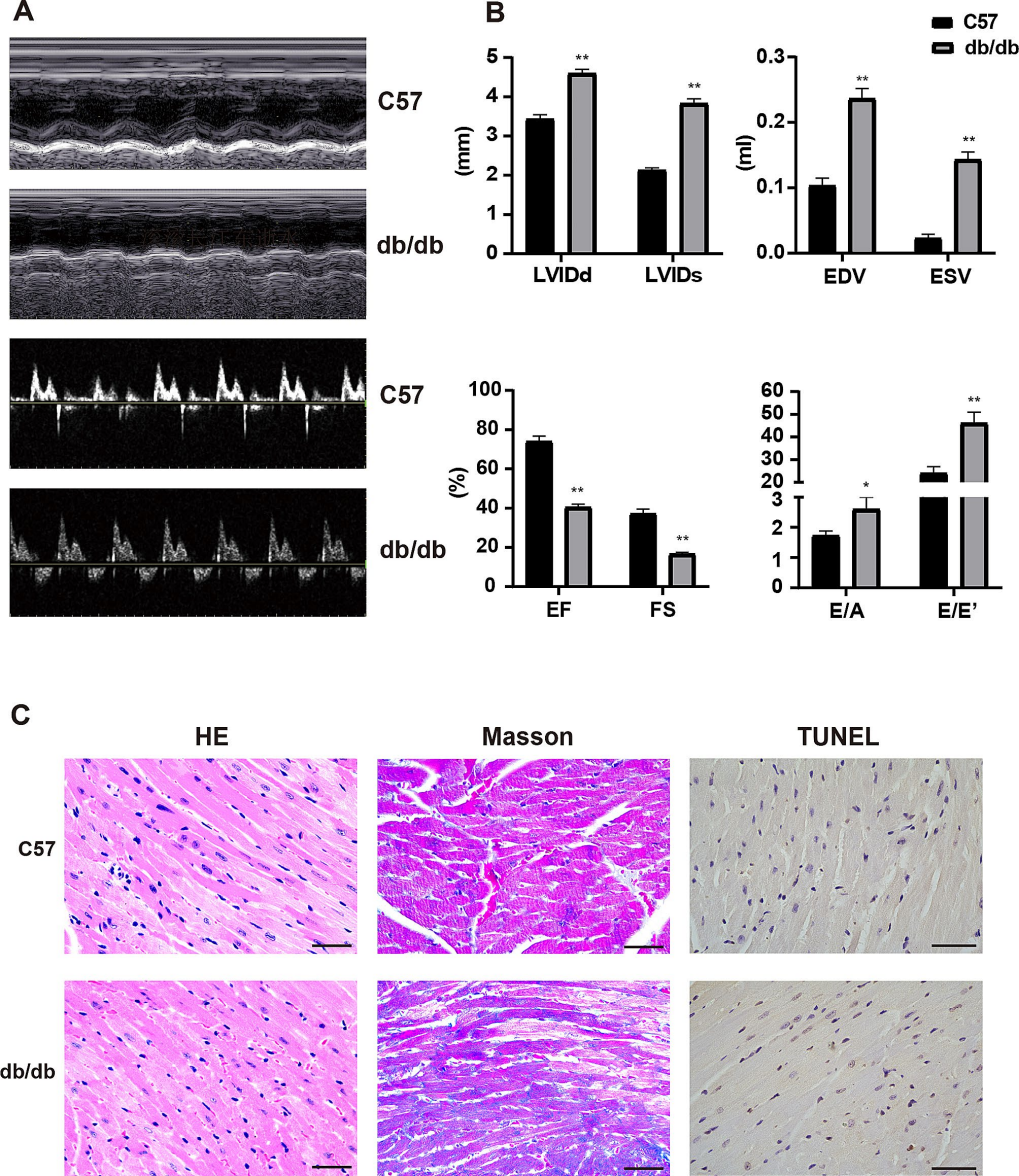
Changes of cardiac function and structure in db/db mice. **(A)** Echocardiographic images showed that the impairment of cardiac systolic and diastolic function in db/db mice compared to C57 mice. **(B)** cardiac parameters hinted a decrease in both systolic and diastolic function in the hearts of db/db mice compared to C57 mice. **(C)** H&E staining images showed disorganized myocardial arrangement in db/db mice. Masson staining images revealed that cardiac collagen fibra were remarkably increased in db/db mice. TUNEL staining indicated that db/db mice have more myocardial apoptosis than C57 mice. Scale bar = 50 μm. Original magnification: 1:400. *n* = 6. Means ± SD were presented. **P < 0.01, *P < 0.05 versus C57 mice

At the age of 20 weeks, LVEDD, LVEDS, EDV, ESV were all increased and LVEF and LVFS were decreased in db/db mice (*p*<0.01. Figure 1A, B). Besides, E/A and E/E’ were dramatically higher in db/db than those in C57BL/6J. In particular, in the db/db mice group with E/A>2, which revealed that severe diastolic dysfunction occurred and cardiac function had been significantly impaired in db/db mice. The histological analysis showed that some of the cardiomyocytes in db/db mice were enlarged with disorders of myocardial cell arrangement compared to control hearts. Masson’s staining displayed massive fibrosis in the diabetic myocardium. TUNEL assay demonstrates myocardial apoptosis were increased in db/db mice compared to C57BL/6J mice (Fig. 1C).

### Expression profiles of lncRNAs and mRNAs in heart tissues

Statistical and rigorous quality control of sequencing data provides a macro view of the quality of library construction and sequencing of samples. Overall, valid data for subsequent analysis were obtained by comparison with the reference genome. The data are shown in Tables 3 and 4.

**Table 3:** Quality control of transcriptome data (*n* = 3)

Sample	Raw Reads	Clean Reads	Q20(%)	Q30(%)
db/db-1	103,878,154	101,563,608	98.39	95.8
db/db-2	112,552,470	109,087,746	98.54	96.05
db/db-3	114,225,348	111,459,532	98.4	95.86
C57-1	94,430,382	91,148,252	98.15	95.48
C57-2	94,177,574	92,021,548	98.37	95.94
C57-3	102,312,792	99,176,202	98.21	95.55

**Table 4:** Mapping statistics of the sequencing reads (*n* = 3)

Sample	Total reads	Total mapped	Multiple mapped	Uniquely mapped
db/db-1	101563608	85453280 (84.14%)	10946031 (10.78%)	74507249 (73.36%)
db/db-2	109087746	95972924 (87.98%)	11727287 (10.75%)	84245637 (77.23%)
db/db-3	111459532	94419869 (84.71%)	13239733 (11.88%)	81180136 (72.83%)
C57-1	91148252	73203256 (80.31%)	10482904 (11.5%)	62720352 (68.81%)
C57-2	92021548	75188607 (81.71%)	11176422 (12.15%)	64012185 (69.56%)
C57-3	99176202	84157959 (84.86%)	9807213 (9.89%)	74350746 (74.97%)

**Fig. 2:**
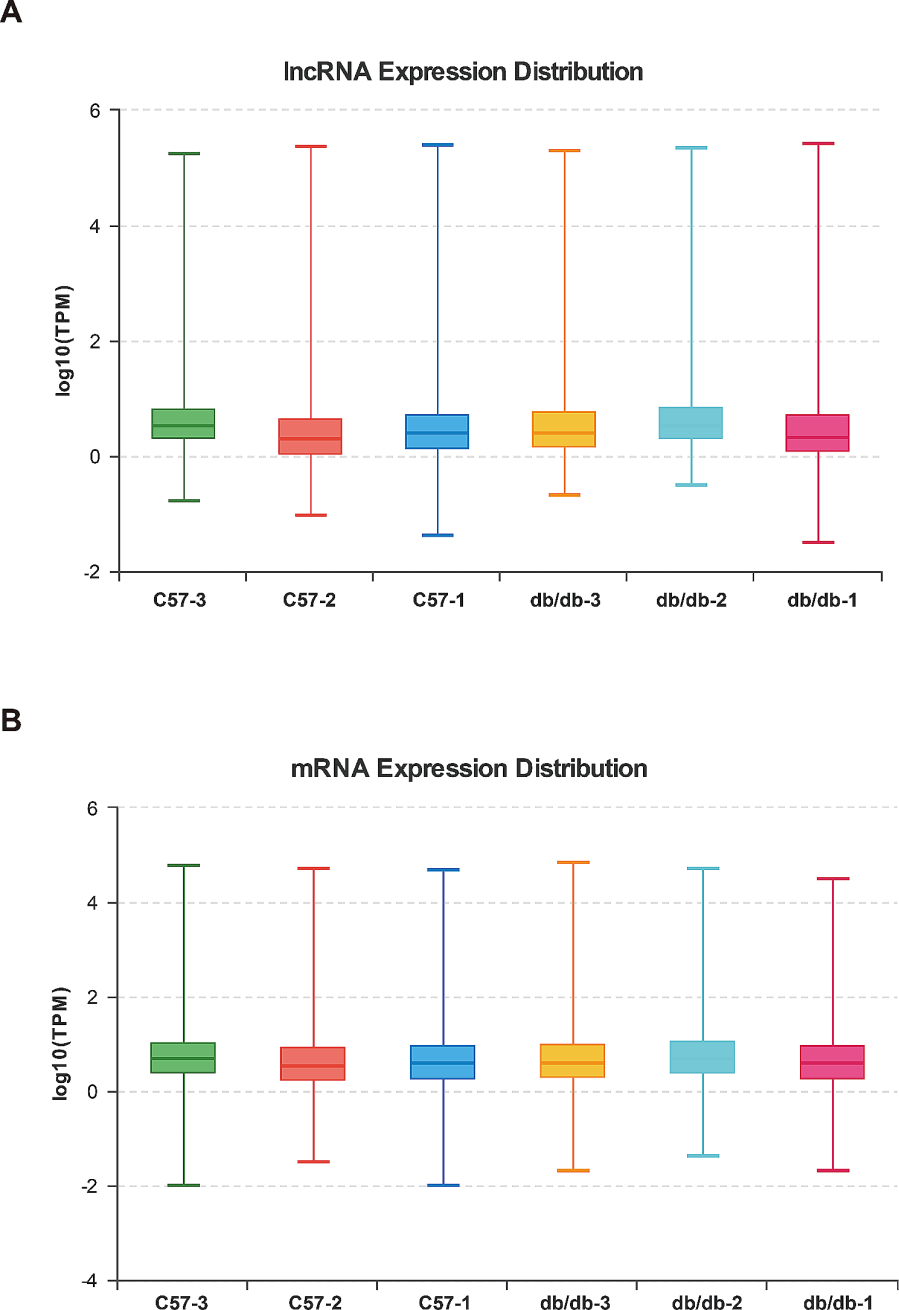
Box plots of expression distribution show similar levels of gene expression in different samples. **(A)** LncRNA expression distribution. **(B)** mRNA expression distribution

**Fig. 3:**
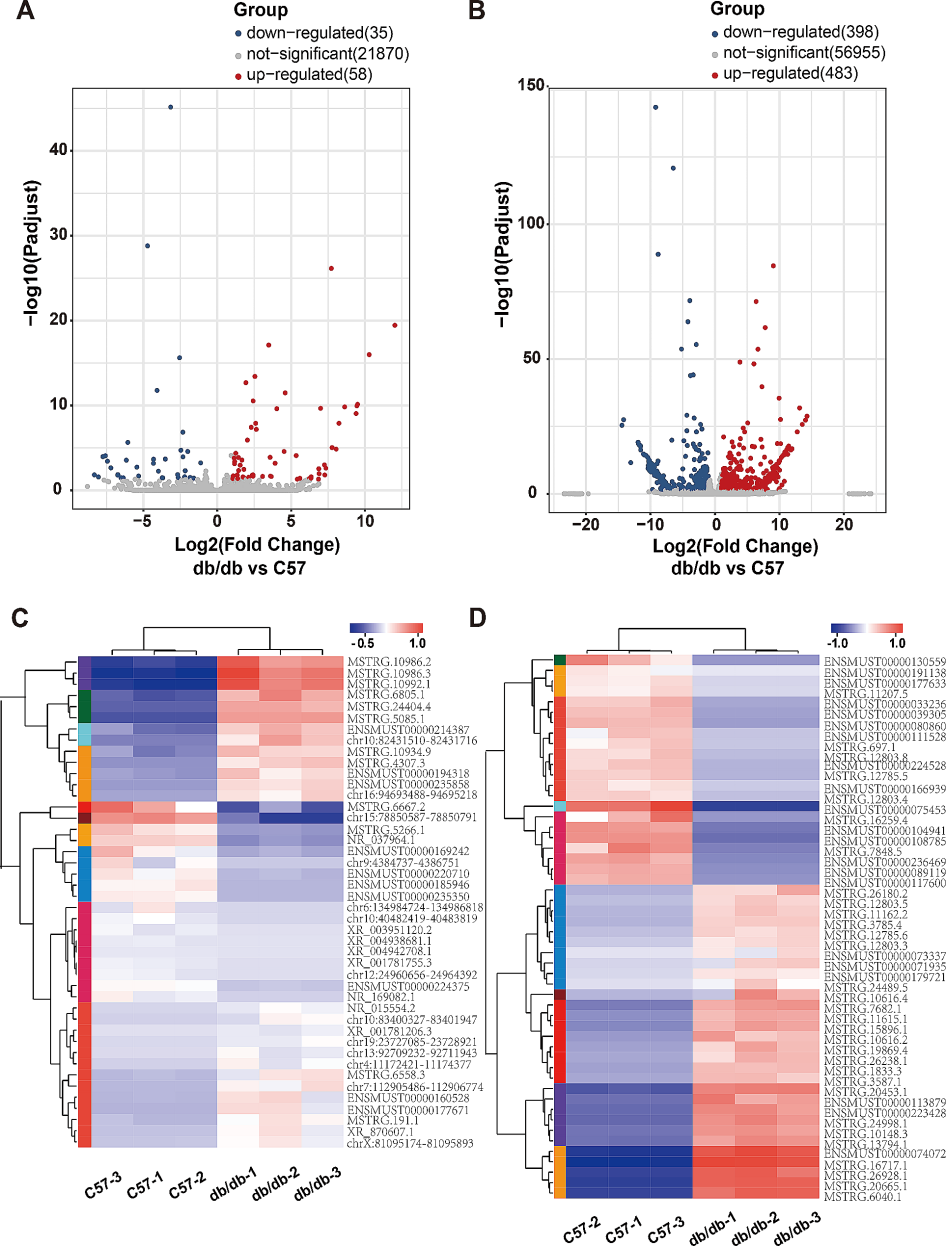
The expression profiles of lncRNAs and mRNAs in myocardium. **(A)** Volcano plot of differentially expressed lncRNAs. **(B)** Volcano plot of differentially expressed mRNAs. Red and blue color represent up- and down-regulated transcripts, respectively. **(C)** Heatmap of partially expressed lncRNAs (|log2FC|>3). **(D)** Heatmap of partially differentially expressed mRNAs (|log2FC|>10). Red color indicates high relative expression levels and blue color indicates low relative expression levels

The lncRNA and mRNA expression patterns in heart tissues between db/db and C57BL/6J mice were found to be significantly different, including a total of 21,963 lncRNAs and 57,836 mRNAs. The box plot showed that the distribution of transcripts and the gene expression levels of different samples in the two groups appeared to be similar (Fig. 2A, B). Next, we found 93 differentially expressed lncRNAs and 881 mRNAs. In db/db mice, the top five up-regulated lncRNAs transcripts were MSTRG.5085.1 (Hmga1b), MSTRG.10992.1 (Gm8909), ENSMUST00000235858 (Gm50252), MSTRG.4307.3 (Cuedc1) and chr16:94693488–94,695,218 (Dyrk1a), and the top five down-regulated lncRNAs transcripts were chr9:4384737–4,386,751 (Msantd4), XR_003951120.2 (4933413J09Rik), NR_169082.1 (Gm41414), ENSMUST00000235350 (2310015A16Rik) and ENSMUST00000185946 (Hand2os1). PCR results showed that ENSMUST00000214387 (Gm48483), ENSMUST00000219113 (Gm47101) and ENSMUST00000060680 (2010001K21Rik) were significantly upregulated, while ENSMUST00000136201 (C030037D09Rik), NR_037964.1 (2610507I01Rik) and chr15:78850587–78,850,791 (Cdc42ep1) were significantly downregulated in heart tissues from diabetic mice, which were consistent with the RNA-sequencing data (Fig. 6C). The volcano plot and heatmap were directly visualized the dysregulated lncRNAs and mRNAs (Fig. 3).

### GO and KEGG analysis

**Fig. 4:**
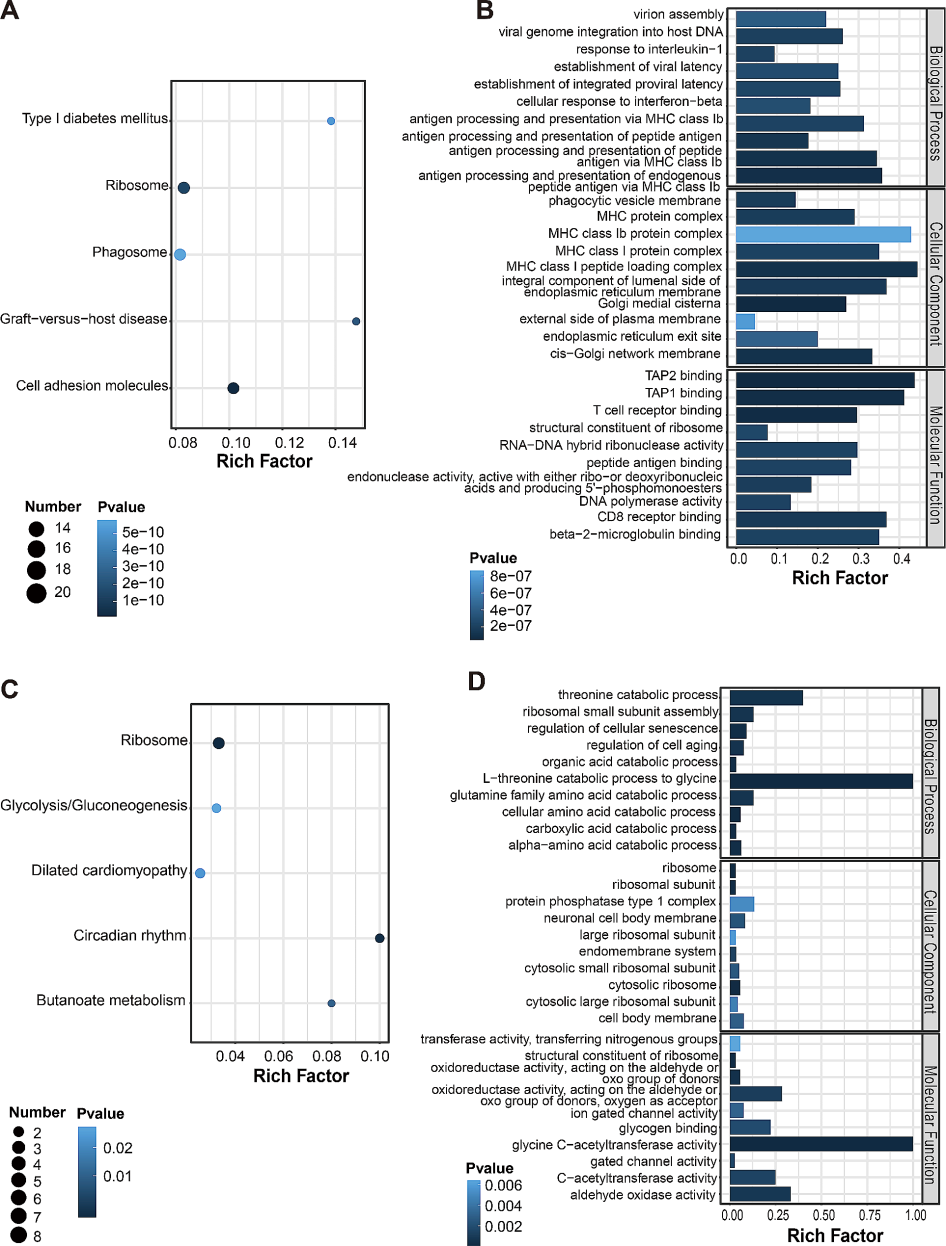
GO and KEGG pathway analysis of differentially expressed genes. **(A)** Top 5 pathways from KEGG pathway analysis of upregulated genes. **(B)** Top 10 terms from GO analysis of upregulated genes. **(C)** The top 5 pathways from KEGG pathway analysis of downregulated genes. **(D)** Top 10 terms from GO analysis of downregulated genes

Up-regulated genes were linked with a total of 896 GO terms, and down-regulated genes were linked with a total of 381 GO terms. The top ten terms of up- and down-regulated genes were shown in Fig. 4B, D. Most of the GO terms are relevant to the immune response and protein catabolism, respectively.

KEGG pathway analysis indicated that 183 and 158 pathways were enriched among the up- and down-regulated genes, respectively. The top 3 pathways that up-regulated genes were significantly enriched in cell adhesion molecules (CAMs), ribosome and graft-versus-host disease. Whereas top 3 down-regulated genes pathways were significantly enriched in ribosome, circadian rhythm and energy metabolism. The pathways were shown in Fig. 4A, C.

### lncRNA-mRNA co-expression network analysis

**Fig. 5:**
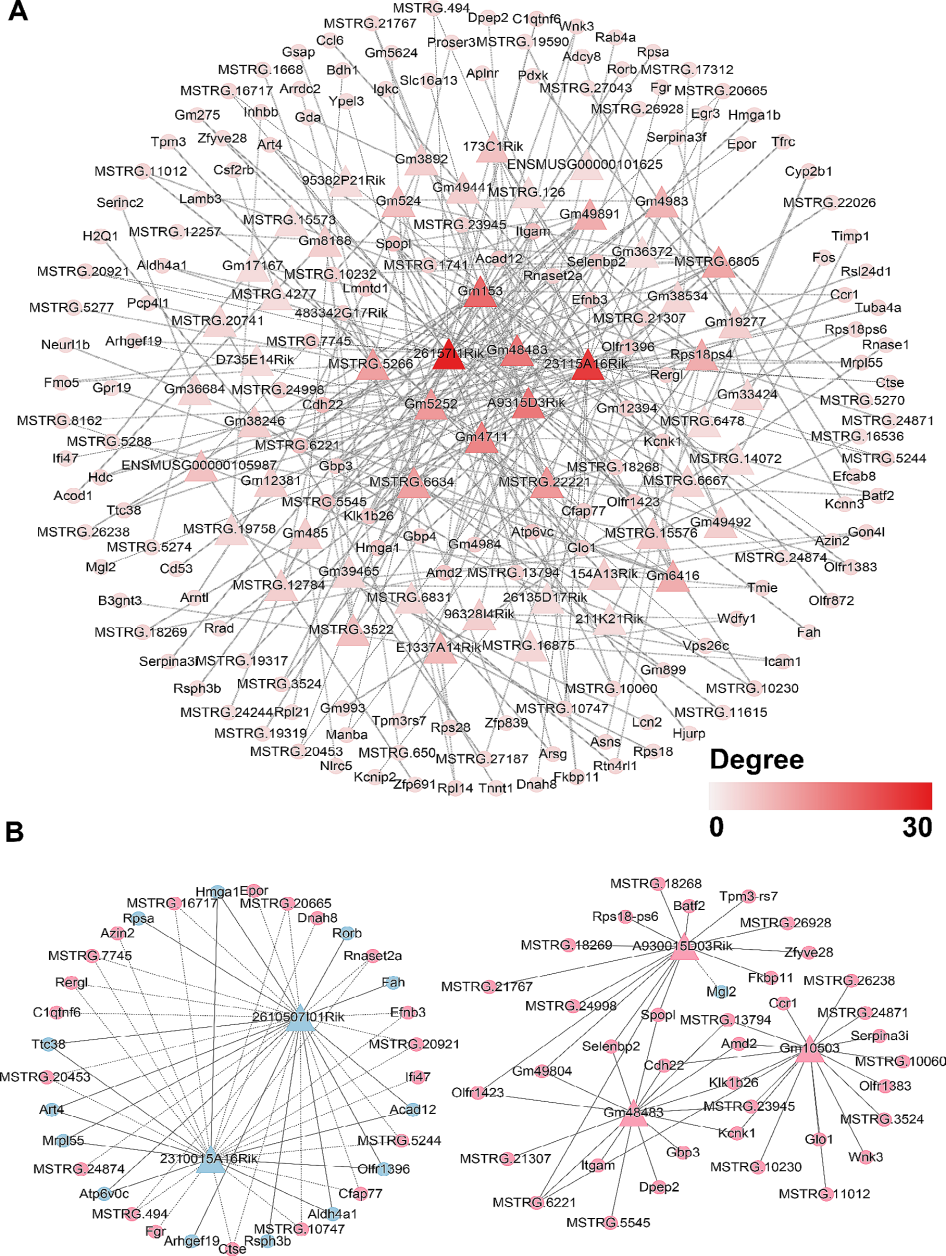
Co-expression network of differentially expressed lncRNAs and mRNAs. **A**. There were 293 connections between 53 lncRNAs and 161 mRNAs in the networks. Triangles and circles represent lncRNAs and mRNAs, respectively. Line type of Vertical Slash for positive correlation, line type of Dash for negative correlation. The shade of the colour represents the node degrees. **B.** Five lncRNAs having maximum connections with mRNAs were taken to construct the co-expression network. In the network, genes colored in pink are upregulated, genes colored in blue are downregulated. Line type of Solid for positive correlation, line type of Equal Dash for negative correlation

**Fig. 6:**
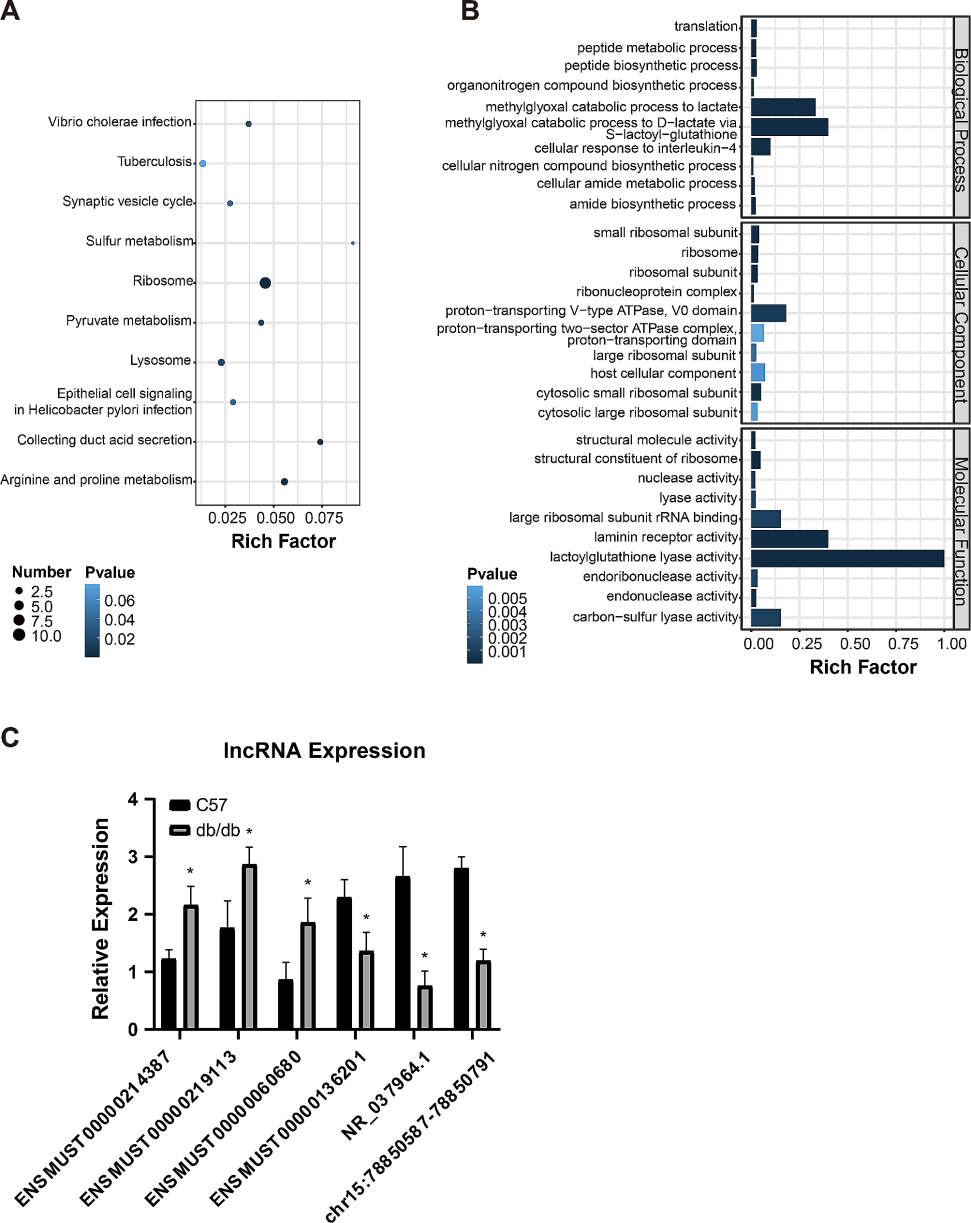
GO and KEGG pathway analysis of genes linked with 5 lncRNAs in the co-expressed network and validation of 6 differentially expressed lncRNAs. **(A)** Top 10 pathways from KEGG pathway analysis of genes linked with 5 lncRNAs in the co-expressed network. **(B)** Top 10 terms from GO analysis of genes linked with 5 lncRNAs in the co-expressed network. **(C)** Validation of 6 differentially expressed lncRNAs (3 upregulated and 3 downregulated) *n* = 3. Means ± SD were presented. *P < 0.05 versus C57 mice

There were 293 connections between 53 lncRNAs and 161 mRNAs in the networks (Fig. 5A). The functions of these differentially expressed lncRNAs were predicted based on GO and KEGG analyses. Subsequently, we filtered 5 differentially expressed lncRNAs that are the most relevant to differentially expressed mRNAs, which were lncRNA 2610507I01Rik, 2310015A16Rik, Gm10503, A930015D03Rik and Gm48483 (Fig. 5B). GO and KEGG enrichment analysis showed that they were involved in the cellular metabolic processes and immune regulation (Fig. 6A, B).

## Discussion

Db/db mice are the most widely used rodent models in the study of T2DM [26–28]. In the present study, db/db mice presented hyperglycaemia, high fat and insulin resistance, displaying the typical features of T2DM. At 20 weeks of age, db/db mice existed severe cardiac dysfunction, and abnormal cardiac structure, including increased myocardial fibrosis and apoptosis. It can be suggested that metabolic disturbances lead to the development of DCM in db/db mice.

A growing body of researches suggest that lncRNAs play a critical role in DCM [29, 30]. It has been reported that the expression of lncRNA MALAT1 are increased in myocardium of db/db mice, and inhibition of MALAT1 attenuates cardiomyocyte apoptosis [22]. Knockdown of lncRNA MIAT can reduce apoptosis and inflammatory responses in cardiac myocytes, and improve cardiac function [21, 31]. Besides, overexpression of lncRNA HOTAIR also improves cardiomyocyte activity in both mice and human [32, 33]. In our study, the expression patterns of lncRNAs in myocardium from diabetic mice were significantly different from those from normal mice, which indicate that some of these genes maybe participate in occurrence of DCM. Some of the molecular mechanisms of DCM may be different in type 1 diabetes mellitus (T1DM) and T2DM, which may contribute to the different expression patterns of certain lncRNAs [34]. In our database, due to the lack of enough samples, some lncRNAs in heart tissues of DCM mice have no significant difference compared to those of the mormal mice, but they were all highly expressed, such as lncRNA H19, GAS5, MEG3, NEAT1 and NORAD. Previous study has shown that lncRNA H19 is elevated in DCM rat hearts, which promote cardiomyocyte apoptosis [35]. Research showed that knockdown of GAS5 protects human cardiomyocytes against HG-induced inflammation by inhibiting miR-21-5p-mediated TLR4/NF-κB pathway [18]. MEG3 and NEAT1 can induce apoptosis of cardiomyocytes under HG condition, respectively [36, 37]. Liu et al. suggest that down-regulated NORAD improves cardiac fibrosis in db/db mice via the NORAD/miR-125a-3p/Fyn axis [38]. In our previous study, we found that lncRNA MALAT1 expression was elevated in HG-induced primary mouse cardiomyocytes and mediated HG-induced apoptosis through activation of the RhoA/ROCK pathway via sponging miR-185-5p [39]. This is consistent with our present results, our RNA-sequencing results showed a trend of increased expression of MALAT1 in DCM group and our data also revealed reduced Bcl-2 expression and elevated Bax expression in db/db mice hearts, which demonstrating a trend toward increased apoptosis in DCM. Zhang et al. found that lncRNA DACH1 was increased in hearts of STZ-diabetic mice and up-regulated DACH1 accelerated cardiomyocyte apoptosis and mitochondrial oxidative stress [40]. However, DACH1 was down-regulated in our DCM mice. These findings provide a valuable and promising therapeutic target for the treatment of DCM. In the present study, the top 10 differentially expressed lncRNAs like Hmga1b, Gm8909, Gm50252, Cuedc1, Dyrk1a, Msantd4, 4933413J09Rik, Gm41414, 2310015A16Rik, Hand2os1 has not yet been reported in myocardium in DCM, which maybe signify that more and more differentially expressed lncRNAs would be found in pathogenesis of DCM with the development of biotechnology.

The results of GO analysis indicated that some up-regulated genes are associated with inflammation, such as response to interleukin-1 (IL-1). IL-1 mediated cardiac inflammation plays a role in DCM [41]. IL-1β is also an important player in the NLRP3 pathway and DCM were aggravated by NLRP3 inflammasome-mediated release of IL-1β and IL-18 [42, 43]. In addition to IL-1β (log2FC = 1.34), the RNA-sequencing results also showed that tumour necrosis factor-α (TNF-α) expression (log2FC = 3.18) was also significantly elevated in heart tissues of DCM mice, which is consistent with our previous findings that levels and gene expression of TNF-α and IL-1β were significantly elevated in serum and myocardial tissues of db/db mice, and they were mediated cardiac inflammation and fibrosis through immune responses [44]. Most of the down-regulated genes are associated with the regulation of cellular metabolic processes and senescence. A few of evidence indicate that the changes in cardiac metabolism play a vital role in pathogenesis of DCM. It is reported that cardiac amino acid catabolism is impaired in DCM mice [3, 45]. In cellular senescence, recent study suggests that aging contributes to the progression of diabetes and its complications [46]. Cell aging increases with age, obesity and diabetes, and is directly related to insulin resistance which plays a vital role in pathology of DCM. Based on the KEGG analyses, these differentially expressed lncRNAs are involved in a variety of biologically pathways, including p53, mTOR, NF-κB, PI3K-Akt, calcium and MAPK signaling pathways. Studies have confirmed that these pathways participate in the pathogenesis of DCM [4]. Upregulation of MALAT1 activates p53 and leads to apoptosis in cardiomyocytes [47]. HOTAIR can improve DCM through regulating the PI3K/Akt pathway [32]. H19 mediates HG-induced apoptosis in cardiomyocytes via regulation of MAPK pathway [35]. Our previous study also showed that the JNK and p38MAPK pathways are associated with HG-induced myocardial apoptosis [48].

Although lncRNAs do not have the same function as mRNAs to directly encode proteins, the functions of lncRNAs may be closely related to their associated protein-coding genes [16, 49, 50]. The co-expression networks are commonly used to reveal the core regulatory lncRNAs [51]. We selected 5 lncRNAs which were the most relevant to differentially expressed mRNAs, including 2610507I01Rik, 2310015A16Rik, Gm10503, A930015D03Rik and Gm48483. These 5 lncRNAs were significantly enriched for protein or amino acid metabolism and immune responses. 2610507I01Rik expression is highly correlated with Atp6v0c and Epor. Hmga1 expression is both associated with 2610507I01Rik and 2310015A16Rik. Studies have shown that in schwann cells of diabetic rats, Epor expression was elevated, leading to a higher rate of apoptosis [52]. Decreased Atp6v0c expression in the gingiva of diabetic mice ultimately exacerbates periodontal inflammation [53]. Low expression of Hmga1 accelerates isoprenaline-induced cardiomyocyte hypertrophy [53]. Although there are currently no detail reports on the above-mentioned genes in DCM, according to GO and KEGG enrichment analysis, these genes would play an important role in the pathological process of DCM, which require further researchers to investigate. Currently, non-coding RNAs are used as a very promising pharmaceutical treatment strategy for a wide range of human diseases at the molecular level [54]. Emerging field of non-coding RNA-based therapies now also attracts attention in cardiovascular studies [55]. Such therapeutic approaches may inhibit molecular function by acting as antagonists, or restore the function of molecules by acting as mimics of endogenous molecules [14].

It must be acknowledged that there are limitations at this stage of our study. This study was limited by its small sample size. Furthermore, regarding some newly discovered lncRNAs that may be involved in the development of DCM, we do not know whether those markers can be used in circulation as indicators to prevent and screen for DCM. Certain circulating miRNAs may serve as biomarkers for a variety of cardiovascular conditions, according to recent research [56]. Like miRNAs, lncRNAs were stably detectable found in plasma. To prevent degradation, circulating lncRNAs may be encapsulated in microparticles such as microvesicles, exosomes, apoptotic microparticles, and apoptotic bodies [57]. Further validation of circulating lncRNAs in humans this would have greater significance for disease screening. It is also important to further validate the mechanistic details of these markers through in vitro and in vivo studies. We will focus on these newly discovered clues in our next efforts, and further experimental studies are needed to verify our findings.

## Conclusions

In conclusion, our results show that db/db DCM mice exist differentially expressed lncRNAs and mRNAs in hearts. According to GO, KEGG and co-expression network analysis, these differentially expressed genes may participate in the process of apoptosis, fibrosis, inflammation, immune response, cellular aging, and abnormal energy metabolism. Our research not only supports the widely recognized pathological changes in DCM, but also provides new ideas and more comprehensive clues for the study of DCM related pathological processes.

## Data Availability

The datasets generated and/or analysed during the current study are available in the SRA repository (Accession number: PRJNA1085532).
